# TGFβ3-mediated induction of Periostin facilitates head and neck cancer growth and is associated with metastasis

**DOI:** 10.1038/srep20587

**Published:** 2016-02-09

**Authors:** Xing Qin, Ming Yan, Jianjun Zhang, Xu Wang, Zongze Shen, Zhongjing Lv, Zhihui Li, Wenyi Wei, Wantao Chen

**Affiliations:** 1 Department of Oral and Maxillofacial-Head & Neck Oncology and Faculty of Oral and Maxillofacial Surgery, Ninth People’s Hospital, Shanghai Jiao Tong University School of Medicine, Shanghai, 200011, China; 2 Shanghai Research Institute of Stomatology and Shanghai Key Laboratory of Stomatology, Shanghai, 200011, China; 3 Department of Pathology, Beth Israel Deaconess Medical Center, Harvard Medical School, 330 Brookline Avenue, Boston, Massachusetts 02215, USA

## Abstract

The matrix-specific protein periostin (POSTN) is up-regulated in human cancers and associated with cancer growth, metastasis and angiogenesis. Although the stroma of cancer tissues is the main source of POSTN, it is still unclear how POSTN plays a role to facilitate the interplay between cancer cells and cancer-associated fibroblasts (CAFs) in head and neck cancer (HNC), thereby promoting tumorigenesis via modifying the tumor microenvironment. Herein, we have performed studies to investigate POSTN and its role in HNC microenvironment. Our results indicated that POSTN was significantly up-regulated in HNCs, especially in the tissues with lymph node metastasis. Moreover, POSTN was highly enriched in the stroma of cancer tissues and produced mainly by CAFs. More importantly, we have pinpointed TGF-β3 as the major upstream molecular that triggers the induction of POSTN in CAFs. As such, during the interaction between fibroblasts and cancer cells, the increased stromal POSTN induced by TGF-β3 directly accelerated the growth, migration and invasion of cancer cells. Hence, our study has provided a novel modulative role for POSTN on HNC progression and further reveals POSTN as an effective biomarker to predict metastasis as well as a potential cancer therapeutic target.

Head and neck cancer (HNC) ranks sixth among malignancies worldwide^1^. Although the living quality of patients with this devastating disease has been raised greatly, the 5-year survival rate of HNC patients has still remained unacceptable throughout the last 3 decades^2^, especially in patients with locoregional lymph node metastases^3^. As HNC severely affects the appearance, swallowing, breathing and psychological state of patients, the application of accurate and efficient biomarker for early diagnosis and prognostic prediction is urgent. Therefore, certain molecules including microRNAs have been defined as potential diagnostic and prognostic biomarkers in advanced HNC^4^,^5^,^6^. It is well known that the initiation and development of HNC is a multi-gene interactive process involving the tumor surrounding microenvironment^7^. Interactions between tumor cells and stromal cells in tumor microenvironment play a very important role in malignant transformation^8^,^9^. Extracellular matrix (ECM), the medium of cell-cell communication in tumor microenvironment, was initially considered as a host barrier against tumor invasion, but evidence shows that ECM could promote tumorigenesis^7^,^10^.

Periostin (POSTN), originally isolated as an osteoblast-specific factor, is a 90-kilodalton secretory protein, which was classified as a component of ECM and functions as a cell adhesive molecule for preosteoblasts^11^. Pertinently, studies have revealed that POSTN could be strongly induced by transforming growth factor-β (TGF-β)^11^, interleukin-4 (IL-4), interleukin-13 (IL-13)^12^, bone morphogenic protein-2 (BMP2)^13^ and platelet derived growth factor-bb (PDGF-bb)^14^. POSTN has become the topic of multiple scientific investigations in different areas since its first identification in 1993^15^. The role of POSTN across health and disease has been integrated and it is actively involved in osteology (bone development and maturation, bone remodeling etc.), cutaneous and connective tissue remodelling, oncology, cardiovascular differentiation, allergic and respiratory diseases, and in various inflammatory settings and diseases^15^,^16^. Considering the functions of POSTN in the development of epithelial–mesenchymal transition (EMT), ECM restructuring and remodelling, many researchers shifted their focus to the role of POSTN in tissue repair and oncology^16^. More recent studies have indicated that the overexpression of POSTN is frequently observed in numerous cancers, including breast cancer, melanoma, colon cancer, gastric cancer etc.^17^. What’s more, high expression of POSTN in cancer cells was reported to be associated with cancer cell growth, invasion, migration, EMT and tumor angiogenesis^17^,^18^,^19^. Recently, studies have shown that POSTN, expressed by fibroblasts in the stroma of the primary and metastatic tumor, is required to allow cancer stem cell maintenance and facilitates cancer cell growth^20^,^21^. Although the up-regulation of POSTN in HNC has been reported^18^, it is still unclear which type of cells, cancer cells or CAFs, are the principal source of POSTN and how this protein plays the linkage role between cancer cells and tumor stroma in HNC.

In this study, we investigated the expression level of POSTN in HNC and demonstrated the functional role of POSTN in the growth and metastasis of HNC cells. Additionally, our data revealed that tumor stroma, especially CAFs, was the important source of POSTN in HNC tissues, and the fibroblast-secreted POSTN created a tumor-supportive microenvironment to facilitate the growth and metastasis of HNC cells.

## Results

### Identification of POSTN as a candidate gene overexpression in HNC

**Figure 1:**
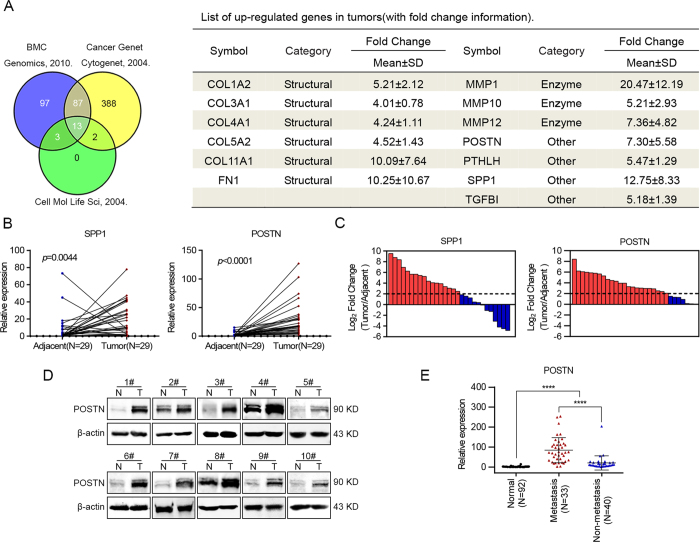
Up-regulation of POSTN in HNC was associated with the metastasis of tumor. (**A**) Venn diagrams and list of the identified up-regulated genes (13 genes) found in three datasets of microarray analysis published by Kuriakose *et al.*^22^, Toruner *et al.*^23^ and Hu *et al.*^24^. (**B**) The expression levels of SPP1 and POSTN in 29 pairs of HNC samples and paired adjacent normal tissues. (**C**) Comparison of SPP1 or POSTN expression levels between HNC samples and their paired adjacent normal tissues. A log2-fold change more than 2 was regarded as significant up-regulation (dotted lines). (**D**) POSTN protein levels were determined in 10 paired HNC samples using western blotting. (**E**) POSTN expression was determined using real-time PCR in 73 cases (involving 33 metastatic cases) of HNC samples and in 92 cases of normal oral epithelial tissues. (****p < 0.0001; N, adjacent normal tissue; T, tumor tissue.)

To understand the nature of genes that are responsible for the pathological progression associated with HNC development, we searched for the microarray data of squamous cell carcinoma from GEO datasets (http://www.ncbi.nlm.nih.gov/gds/) to identify candidate genes that are highly expressed in HNCs. Three studies were selected: first, an analysis of 22 paired normal tissues and tumor samples from patients with HNC^22^; second, an analysis of 16 cases of oral squamous cell carcinoma tissues and 4 cases of normal tissues^23^; and third, an analysis of 17 paired esophageal squamous cell carcinoma samples and adjacent normal tissues^24^. Among the mutual dysregulated genes of the three publications, 13 genes (fold change ≥3 and *p* < 0.05) were increased in the tumor tissues (Fig. 1A).

Of these genes, 6 were predicted to be ECM structural constituents, 3 belonged to matrix metalloproteinases, and the other 4 were identified as being involved in “ECM organization,” “cell adhesion,” and the “extracellular region” pathways (Fig. 1A). It has become evident that ECM could promote tumor growth and metastasis^7^. Apart from the transforming growth factor-beta-induced protein (TGFBI) which is a secreted ECM component that can play an important role in tumor progression^25^,^26^, POSTN, parathyroid hormone-like hormone (PTHLH) and secreted phosphoprotein 1 (SPP1) were also predicted to be associated with skeletal system development. Our research group have previously reported that PTHLH can act as a tumor promoter, affecting cell proliferation and the cell cycle in HNC^27^. Thereafter, the expression levels of POSTN and SPP1 were tested in 29 paired HNC samples through real-time PCR. The data revealed that the POSTN and SPP1 expression levels were increased in HNC tissues (Fig. 1B). SPP1 was overexpressed in 58.6% (17 of 29) of these HNC cases, while POSTN was found to be up-regulated in 79.3% (23 of 29) of the HNC cases (Fig. 1C). Compared with the expression levels of SPP1 in HNC tissues, POSTN was more suitable to be regarded as a candidate gene for overexpression in HNCs.

### Overexpression of POSTN in HNC clinical samples

Western blotting was performed to investigate the protein levels of POSTN in HNC tissues and the results indicated that POSTN was highly expressed in cancer samples compared with adjacent normal tissues (Fig. 1D). Correspondingly, semi-quantitative RT-PCR analyses showed that the mRNA levels of POSTN were also remarkably up-regulated in 18 cases of HNC tissues (Supplementary Figure S1A). The POSTN expression levels were further investigated in expended HNC cohorts using real-time PCR. Consistent with the above data, POSTN was found to be up-regulated in 73 cases of HNC samples compared with 92 cases of normal oral epithelial tissues (Fig. 1E). Meanwhile, the correlations between POSTN mRNA levels and clinicopathological parameters of the patients with HNC were assessed, respectively. As shown in Fig. 1E and in Table 1, POSTN was found to be associated with lymph node metastasis (*p* = 0.018). However, no significant association was determined between POSTN expression level and other parameters. Collectively, these findings suggested that the gain of POSTN expression may contribute to the metastasis of HNC.

### POSTN is produced by fibroblasts instead of cancer cells

**Figure 2:**
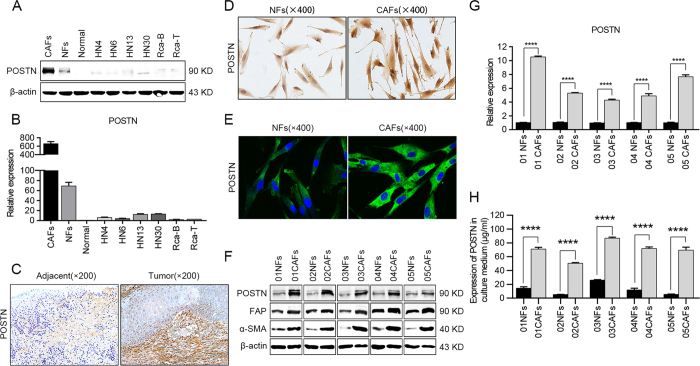
CAFs contributed to the overexpression of POSTN in HNC tissues. (**A,B**) The transcriptional and translational statuses of POSTN were determined in CAFs and NFs, 6 representative HNC cell lines and normal oral epithelial cells (titled normal) using western blotting (**A**) and real-time PCR (**B**). (**C**) Representative images of immunohistochemical staining showed the distribution of POSTN expression in adjacent normal tissues and HNC tissues. (**D,E**) POSTN expressions in NFs and CAFs were detected using immunohistochemical staining (**D**) and confocal microscopy (**E**). (**F**) The protein levels of POSTN, FAP and α-SMA were determined by a western blot analysis. (**G**) POSTN mRNA levels in 5 pairs of NFs and CAFs were measured by real-time PCR. (**H**) POSTN protein levels in the media of NFs and CAFs were determined by ELISA analyses. (****p < 0.0001).

We also analyzed the expression of POSTN in the cell lysates from 6 HNC cell lines (HN4, HN6, HN13, HN30, Rca-B and Rca-T), normal oral epithelial cells and fibroblasts (NFs and CAFs) isolated from paired HNC tissues by western blot analyses. However, the expression of POSTN was found to be slightly increased in HNC cells compared to normal oral epithelial cells. When compared with the NFs and CAFs, the expression levels of POSTN in HNC cells were so low that they were ignored (Fig. 2A). Similar results were also obtained by real-time PCR (Fig. 2B) and semi-quantitative RT-PCR analyses (Supplementary Figure S1B) when investigating the mRNA levels of POSTN in these samples. These data suggested that cancer cells were not the principal source of POSTN in HNC tissues.

To further determine the main source of POSTN, immunohistochemical staining was implemented to detect the distribution of POSTN expression. Notably, we observed that POSTN was mainly expressed in the stromal cells of HNC tissues and adjacent normal tissues, and the expression of POSTN in the stroma of cancer tissues was much higher (Fig. 2C). Then, NFs and CAFs were isolated from adjacent normal tissues and HNC tissues, respectively. The results of immunohistochemical staining (Fig. 2D) and confocal microscopy imaging (Fig. 2E) showed that POSTN was highly expressed in CAFs and slightly expressed in NFs. To further verify the accuracy of our data, western blotting, real-time PCR and semi-quantitative RT-PCR analyses were performed to detect the POSTN expression in another 5 paired NFs and CAFs, and same results were obtained (Fig. 2F,G and Supplementary Figure S1C). Among them, α-SMA and FAP, regarded as CAF-specific markers in previous study^28^, were both highly expressed in CAFs (Fig. 2F). As POSTN is a secreted protein and also functions as a secreted protein, higher expression levels of POSTN were found in the culture medium of CAFs by ELISA analysis (Fig. 2H). Hence, we believed that it was the cancer stroma, especially CAFs, that functioned as the principal source of POSTN in HNC tissues.

### Co-culture with HNC cells induces the POSTN expression level in NFs

**Figure 3:**
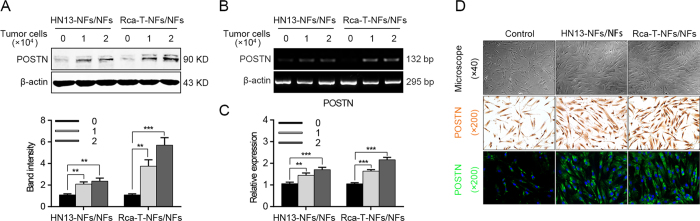
POSTN expression levels increased in NFs during co-culture with HNC cells. (**A**) The POSTN protein was up-regulated in NFs after co-culture with HNC cells as determined by a western blotting. (**B,C**) The POSTN mRNA levels were increased in NFs after co-culture with HNC cells as detected using semi-quantitative RT-PCR and real-time PCR. (**D**) The morphological change of NFs observed by a biological microscopy and the POSTN expression levels in NFs were determined using immunohistochemical staining and confocal microscopy after the co-culture with HNC cells. (**p < 0.01; ***p < 0.001).

Because POSTN was expressed in CAFs from cancer tissues, but was weak in cancer cells, we hypothesized that the interplay between fibroblasts and cancer cells might contribute to the high expression of POSTN. Hence, NFs were co-cultured with HN13 and Rca-T cells, respectively. A significant overexpression of POSTN was observed in NFs after co-culture (Fig. 3A–C), but not in HNC cells (data not shown). Interestingly, with more HNC cells added into the co-culture system, higher expression levels of POSTN would be detected in NFs (Fig. 3A–C). Moreover, we found morphologically that short shuttle-shaped NFs were changed into elongated spindle-shaped NFs after co-culture with HNC cells. In addition, a spiral growth mode was observed and an increased expression of POSTN was detected in the co-cultured NFs through immunohistochemical staining and confocal microscopy observation (Fig. 3D). These data revealed that HNC cells were sufficient to trigger the expression of POSTN in NFs when they were seeded in a co-culture system.

### Exogenous POSTN accelerates the proliferation, migration and invasion of HNC cells

**Figure 4:**
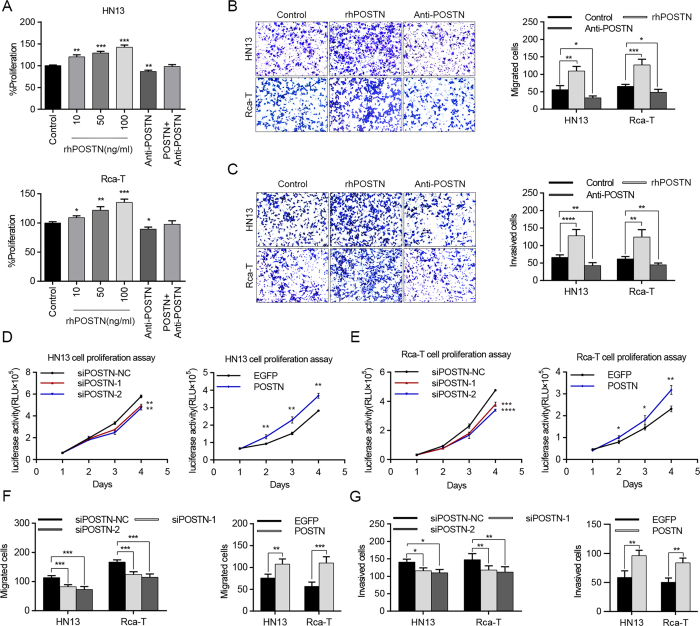
Functional analyses of POSTN effects on HNC cell proliferation, migration and invasion. (**A**) Recombinant human POSTN increased the proliferation of HN13 and Rca-T in a dose-dependent manner, while neutralization of POSTN (100 ng/mL) with anti-POSTN (50 ng/mL) antibody inhibited the proliferation of HN13 and Rca-T. (**B,C**) Recombinant human POSTN (100 ng/mL) promoted the migration and invasion of HNC cells (HN13 and Rca-T), while neutralization of POSTN inhibited the migration and invasion of HNC cells (HN13 and Rca-T). (**D,E**) POSTN knockdown in CAFs partially inhibited the growth of HN13 and Rca-T, while POSTN overexpression in NFs facilitated proliferation of HN13 and Rca-T. (**F,G**) POSTN knockdown in CAFs inhibited the migratory and invasive abilities of HN13 and Rca-T; POSTN overexpression in NFs increased the migration and invasion of HN13 and Rca-T cells. (*p < 0.05; **p < 0.01; ***p < 0.001).

Exogenous recombinant human POSTN (rhPOSTN) was used to imitate the function of POSTN protein in the proliferation and metastasis of HNC cells. HN13 and Rca-T cells proliferated significantly in a dose-dependent response to rhPOSTN more than control cells, while the growth of HN13 and Rca-T cells was inhibited when treated with POSTN antibody. Furthermore, rhPOSTN could partly attenuate POSTN antibody-induced growth inhibition (Fig. 4A). Simultaneously, rhPOSTN drastically facilitated the migration and invasion of HN13 and Rca-T cells, while POSTN antibody partly decreased the migration (Fig. 4B and Supplementary Figure S2) and invasion (Fig. 4C) of cancer cells.

To assess the effect of POSTN expression levels in fibroblasts on the proliferation of cancer cells, fibroblasts of different POSTN expression were co-cultured with the luciferase-expressing HNC cells and the cell number was quantified by measuring the luciferase activity of the co-culture system. We found that silencing of POSTN by siRNA in CAFs (Supplementary Figure S3A) could partially decrease the growth of HN13 and Rca-T cells (Fig. 4D,E). Meanwhile, exogenous POSTN expression in NFs (Supplementary S3B) would facilitate the proliferation of HN13 and Rca-T cells (Fig. 4D,E). Similar results (Supplementary Figure S4) were observed when these fibroblasts were co-cultured with other HNC cells (HN4, HN6, HN30 and Rca-B). In addition, CAFs transiently transfected with siRNA-POSTN had a decreased effect on the migration and invasion of HNC cells, while NFs transfected with POSTN-expressing plasmid would promote the migration and invasion of HNC cells (Fig. 4F,G, Supplementary Figure S5). Therefore, we concluded that POSTN created a tumor-supportive microenvironment for the progression of HNCs.

### The soluble POSTN acts on HNC cells by binding the integrin receptor

**Figure 5:**
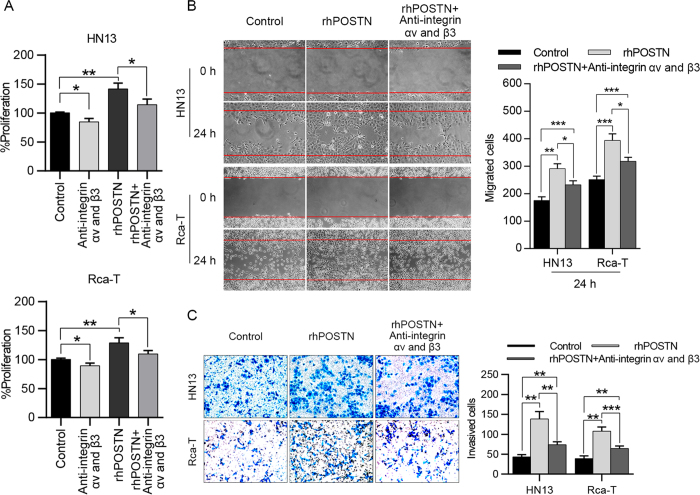
POSTN exhibited its effects on HNC cells by binding an integrin receptor. (**A**) The mixture of anti-αv (5 μg/mL) and anti-β3 (5 μg/mL) integrin antibodies partially decreased the POSTN-inducing (100 ng/mL) proliferation of HN13 and Rca-T cells. (**B,C**) Recombinant human POSTN (100 ng/mL) promoted the migration and invasion of HNC cells (HN13 and Rca-T), while blocking of αv and β3 integrins by integrin antibodies (5 μg/mL of each) inhibited the migration and invasion of HNC cells (HN13 and Rca-T) cultured in DMEM with 100 ng/mL POSTN. (*p < 0.05; **p < 0.01; ***p < 0.001).

Integrin αvβ3, αvβ5 and α6β4, well-known receptors for POSTN, are thought to be involved in tumorigenesis. Recent advances have revealed that POSTN could act as a ligand for αvβ3 and αvβ5 integrins to inhibit the invasion and anchorage-independent growth of HNC cells^18^. In this study, we found that interference with the function of integrins by specific anti-αv and anti-β3 integrin antibodies could partially decrease the effects of the soluble POSTN on the proliferation (Fig. 5A), migration (Fig. 5B) and invasion (Fig. 5C) of HNC cells. Hence, we presumed that CAF-derived POSTN played its role in tumor progression partly by binding an integrin receptor.

### TGF-β3 acts as a regulator in POSTN expression

**Figure 6:**
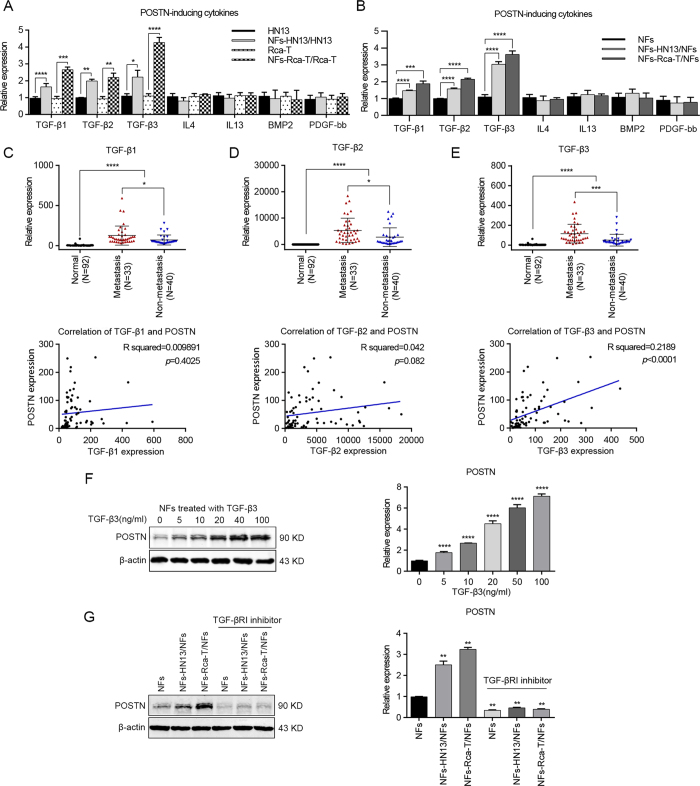
TGF-β3 induced POSTN expression in NFs after co-culture with HNC cells. (**A,B**) The mRNA levels of TGF-β1, TGF-β2, TGF-β3, IL-4, IL-13, BMP2 and PDGF-bb in HNC cells (**A**) and NFs (**B**) were detected after the co-culture of NFs and HNC cells by real-time PCR. (**C–E**) TGF-β1, TGF-β2 and TGF-β3 mRNA levels were determined using real-time PCR in 73 cases of HNC tissues (involving 33 metastatic cases) and in 92 cases of normal oral epithelial tissues. A significant positive correlation was observed between the TGF-β3 and POSTN expression levels in the HNC tissues (n = 73). (**F**) TGF-β3 induced POSTN expression in NFs in a dose-dependent manner. Protein expression by western blot analysis and mRNA expression by real-time PCR were measured at the indicated concentrations. (**G**) Blocking of TGF-β3 with the inhibition of type I TGF-β receptor (SB431542, 10 μM) abrogated the induced-POSTN expression in NFs after the co-culture of NFs and HNC cells. POSTN protein and mRNA levels were determined in NFs using western blotting and real-time PCR. (*p < 0.05; ** *p* < 0.01; ***p < 0.001; ****p < 0.0001).

As noted above, TGF-β1, TGF-β2, TGF-β3, IL4, IL13, BMP2 and PDGF-bb are soluble inducers of POSTN. To investigate the relationship between these soluble cytokines and POSTN expression, NFs were co-cultured with HN13 or Rca-T cells for 72 hours. Interestingly, we found that TGF-β1, TGF-β2 and TGF-β3 mRNAs were all remarkably up-regulated in the co-cultured cancer cells (Fig. 6A) and NFs (Fig. 6B) using real-time PCR, whereas IL-4, IL-13, BMP2 and PDGF-bb mRNAs were not affected in these cells (Fig. 6A,B).

Further investigations indicated that the mRNA levels of TGF-β1, TGF-β2 and TGF-β3 were increased in HNC tissues and positive associations with lymph node metastases were obtained (Fig. 6C–E). Furthermore, correlation analysis showed that there was a prominent positive correlation between POSTN and TGF-β3 (Pearson’s correlation R squared = 0.2189, *p* < 0.0001) mRNA levels (Fig. 6C–E). Meanwhile, adding recombinant human TGF-β3 to NFs could significantly up-regulate the expression of POSTN in NFs with a dose-dependent effect (Fig. 6F and Supplementary Figure 6A). In addition, the inhibition of TGF-β signaling via sufficient type I TGF-β receptor significantly blocked the co-culture inducing POSTN expression in NFs (Fig. 6G and Supplementary Figure 6B). These data demonstrated that TGF-β3 was a key regulator of POSTN expression in NFs during the co-culture with HNC cells.

### Stromal cell-derived POSTN promotes tumor growth and is associated with metastasis *in vivo*

**Figure 7:**
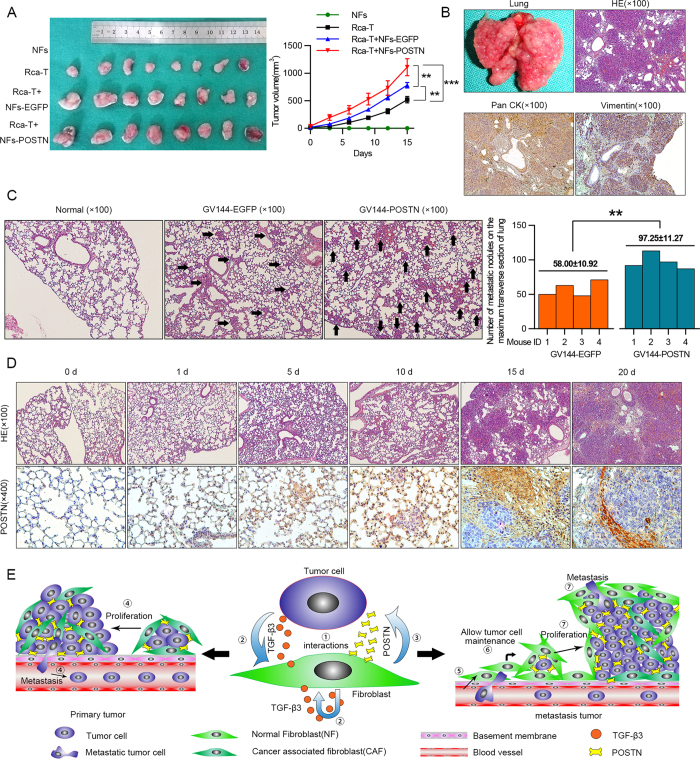
Functional analysis of induced-POSTN effects on xenograft tumor growth and cancer cell lung colonization. (**A**) Exogenous POSTN expression in NFs promoted xenograft tumor growth of Rca-T cells. Tumors’ volumes were measured every third days (mean ± SD) and the tumor growth curve was plotted finally. (**B**) The Rca-T cells (1 × 10^6^) were injected into the lateral tail vein, and hematoxylin and eosin (H&E) staining showed that the pulmonary metastases were poorly differentiated squamous cell carcinoma. Immunohistochemical staining was used to determine the expressions of Pan CK and Vimentin in metastatic loci. (**C**) Number of micrometstatic leisions on the maximum transverse section of the lungs from mice (n = 4) 10 days after tail vein injection with POSTN-expressing Rca-T cells (1 × 10^6^), mean nodules per transverse section values was shown (right). Representative hematoxylin and eosin (H&E) staining of lung tissues with micrometastatic nodules were shown (left). (**D**) There was a time-dependent POSTN expression in lung stromal cells around the pulmonary metastases after the Rca-T cells (1 × 10^6^) were injected into the lateral tail vein. (**E**) A model illustrating the novel modulative role of fibroblast-derived POSTN in controlling tumor growth and metastasis.

After evaluating the relationship between POSTN and tumor progression *in vitro*, we next analyzed whether the induced POSTN affected tumorigenicity *in vivo* using an Rca-T xenograft model in the BALB/C nude mice. As shown in Fig. 7A, NFs could trigger xenograft tumor growth of Rca-T cells and the POSTN-expressing NFs exhibited more significant stimulative effects on the xenograft tumor growth. Rca-T cells, lung metastatic rate of which was 100%, were used to establish an animal model of experimental lung metastasis. The surface of the lung was covered in nodules of metastatic tumors, which were pathologically diagnosed as poorly differentiated squamous cell carcinoma (Fig. 7B). The *in vivo* effects of the secreted POSTN on metastasis were then evaluated. Rca-T cells and POSTN-expressing Rca-T cells were injected into the tail vein of immunodeficient mice. After 10 days, histological analyses confirmed that the number of micrometastatic lesions on the maximum transverse section was markedly increased in the lungs of mice (n = 4) injected with POSTN-expressing Rca-T cells (Fig. 7C). As shown in Fig. 7D, weak staining of POSTN was found in normal lung tissues of mice. Interestingly, the increased expression of POSTN was observed in the stromal cells around the tumor nodules in a time-dependent manner after Rca-T cells were implanted in the lung niche (Fig. 7D). Besides promoting tumor progress, our data indicated that the induced-POSTN might be involved in the local engraftment of metastatic HNC cells. Overall, the induced-expression of POSTN in the ECM allowed for foreign cancer cell maintenance and facilitated the growth, migration and invasion of HNC cells (Fig. 7E).

## Discussion

The matrix-specific protein POSTN plays a functional role in the regeneration of tissues such as bone and heart, and promotes wound healing and tumor invasion^29^. Consistent with previous studies^18^,^19^, our data showed that POSTN was up-regulated in HNC and correlated with the tumor cell growth and metastasis. Higher expression of POSTN was observed in HNC tissues of the patients with lymph node metastasis, confirming that overexpression of POSTN might contribute to tumor malignancy, especially the metastatic potential.

The tumor microenvironment, a cellular environment in which the tumor exists, is composed of tumor cells, surrounding blood vessels, immune cells, CAFs, myeloid-derived suppressor cells, ECM and other secreted signaling molecules. For tumors, the close correlation and constant interactions between the tumor and the surrounding microenvironment are necessary for its existence and progression, similar to the hypothesis of “seed and soil.” Many of the signals that drive the proliferation and invasion of tumor cells were reported to originate from the stromal cell component of the tumor mass^30^,^31^. As the predominant component of stroma, quiescent resident fibroblasts are transformed into CAFs after the interaction with tumor cells. The promoting role of CAFs in tumor progression has been unambiguously elucidated^32^. It is reported that chemokines and cytokines, secreted by CAFs, could facilitate tumor progression by stimulating receptor tyrosine kinase signaling and EMT programs^32^. Moreover, evidence shows that CAFs could also secrete some distinctive ECM to accelerate the attachment, anchorage-growth and metastasis of tumor cells^33^. However, it is still unclear which component of CAF-derived ECM could help to propel the malignant progression of tumors.

As mentioned above, POSTN was predominantly found to be distinctively localized in the stroma of HNC tissues. Although our data verified that the expression of POSTN was slightly increased in tumor cells, CAFs were demonstrated to be the principal source of POSTN. We believe that POSTN derived from HNC cells could only partially account for the tumor-promoting role of POSTN in tumor malignancy, which suggests that CAF-derived POSTN might be fundamental to many control processes in HNC microenvironment. Nevertheless, some published studies indicated that CAFs may actually suppress the tumor progression^34^,^35^. Considering the sophisticated interactions between CAFs and tumor cells, further studies should be performed to reveal the mysterious nature of CAFs.

In the present study, TGF-βs, especially TGF-β3, was found to be associated with the POSTN expression in CAFs during the co-culture with tumor cells. It is well known that TGF-β regulates cell growth and differentiation and is a major inducer of EMT in cancer progression, especially in metastasis^36^. Therefore, people may question whether the increased POSTN is induced by activation of TGF-β signaling pathway rather than a concomitant phenomenon, as TGF-β3 could promote EMT in cancer cells directly to facilitate tumor metastasis. Obviously, we have analyzed our data and reported studies to answer the questions. First, gain of secreted POSTN proteins in culture medium could promote the proliferation and metastasis of HNC cells and vice versa. In addition, the currently available data indicate that the administration of acute low doses of exogenous TGF-β3 is unlikely to influence tumor initiation or progression, and TGF-β3 may actually play a protective role against tumorigenesis^37^. Last but not least, we have identified TGF-β3 by screening a panel of cytokines as the key regulator of POSTN expression in tumor microenvironment. Although TGF-β3 is likely to propel the metastasis of tumor cells to some extent, it is undisputed that TGF-β3 could induce POSTN expression during the co-culture.

A recent study revealed that infiltrating tumor cells need to induce stromal POSTN expression in the secondary target organ to support metastatic colonization and initiation, and the induced POSTN secreted by CAFs in the stroma of the metastatic loci was required to allow for the maintenance of cancer stem cells^21^. Simultaneously, the metastatic colonies were strongly diminished in POSTN-deficient animals compared with the wild-type^21^. What mentioned above indicated that the induced POSTN in stroma might modulate the tumor microenvironment for the metastatic tumor cells to initiate colonization. However, POSTN was also regarded as a potential contributor to tumor fibrillogenesis, which might act as a suppressor of invasion and metastasis in the malignance of some human cancers^29^, such as bladder cancer^38^. Biphasic effects of POSTN on the invasiveness were found in human pancreatic cancer by Erkan *et al.*^39^. Just as their results indicated^39^, the induced POSTN obviously increased the invasiveness of pancreatic cancer cells up to 1.9- and 3.0-fold at a concentration of 100 ng/ml, while the invasiveness decreased at 1 μg/ml. Accordingly, we concluded that POSTN might be associated with ECM remodeling, and the matrix stiffness related to the concentration of POSTN was involved in the suppression of invasiveness.

The integrin-mediated adhesion to POSTN was reported to form a structure of podosome, which was required for or facilitated migration of many cancer types^40^. But excess podosomes could be formed under higher concentration of POSTN and the cells would get “too stuck” to migrate well. This may surely be a partial explanation for the conflicting results. In addition, we have verified that POSTN derives from stromal cells is more than that from epithelial cells including tumor cells of epithelial origin, and evidence has shown that tumor cells of mesenchymal origin could secrete higher concentration of periostin. Thus, it may be that POSTN could optimally support or increase cell motility at an intermediate concentration while a higher amount may be suboptimal. Therefore, we believe that the conflicting impact of CAF-derived POSTN in tumor progression may be partially due to the different tumor types and the different origins of tumors, and further studies are needed to address if and how podosome formation is associated with motility on periostin.

Apart from POSTN, our existing data indicate that TGFBI and SPP1 are overexpressed in HNC tissues. TGFBI, a secreted ECM protein, was reported to have dual action in cancer, functioning as tumor suppressor or promoter^41^. There are more causative evidences pointing to the controversial role of TGFBI in carcinogenesis, but multiple publications report a causative tumor promoting function of TGFBI^42^. TGFBI was found overexpressed in numerous tumors and was reported to be associated with tumor growth, metastasis and poor prognosis^42^,^43^. In HNC, TGFBI was validated to be up-regulated in tumor samples, which could promote tumorigenesis^44^. However, there were few evidences demonstrating that TGFBI would facilitate HNC cell growth, motility or invasiveness. POSTN and TGFBI, both TGF-β-induced matricellular proteins, are paralogs, have some over-lapping functions and act as ligands for integrin receptors^45^. Maybe, TGFBI would have the same activities in their system as POSTN does. However, we could just conclude that TGFBI is up-regulated in HNC and acts as a promoter in tumor progression. There is much to be done to reveal the potential synergy between POSTN and TGFBI. In addition, SPP1, also known as osteopontin (OPN), is an ECM protein and always involved in a series of physiological and pathological processes^46^. Recent studies reported that SPP1 overexpression had been found in numerous cancers including HNC, breast cancer, lung cancer, gastric cancer, hepatocellular carcinoma, colorectal cancer etc.^47^. The elevated SPP1 levels in cancer cells are associated with cellular proliferation, invasion and angiogenesis^48^. In HNC, SPP1 has been reported to have prognostic impact where high SPP1 plasma levels always led to worse outcome^49^,^50^. Irradiation could induce the SPP1 expression in HNC cells and silencing SPP1 expression often resulted in enhanced radiosensitivity^50^. Furthermore, hypoxia could trigger HNC cell line to express more SPP1^51^. Increasing evidences indicate that SPP1 is a prognostic marker for many tumors, but few studies have focused on the role of SPP1 in tumorigenesis and metastasis of HNC. Hence, further studies should be taken to investigate the functional role of SPP1 together with the synergy between POSTN and SPP1 in HNC progression.

In this study, we used two siRNAs (siPOSTN-1 and siPOSTN-2) to knock down POSTN expression in CAFs. POSTN has nine transcript variants and what’s interesting was that we found siPOSTN-1 could knock down four splice variants and siPOSTN-2 nine variants. The data showed that siPOSTN-2 had a better siRNA efficiency than siPOSTN-1 and a significant difference was detected between them. As for the effects of POSTN-silenced CAFs knocked down by siPOSTN-1 and siPOSTN-2 on the biological behaviors of tumor cells, only part of the experimental results was statistically significant, although siPOSTN-2 sometimes had a greater effect. It may be because that the concentration of POSTN varied in small scale barely set influences on tumor cells after the knock-down of POSTN in CAFs by siRNAs. However, our existing results could not rule out that the different siRNA efficiency and effect of siPOSTN-1 and siPOSTN-2 might partially due to the different number of splice variants the two siRNAs can target. In future study, it would be more interesting if we could determine which variants are expressed in NFs and CAFs, and if they have differential activity.

Taken together, POSTN was upregulated in HNCs, especially in the tissues with lymph node metastasis. Moreover, POSTN was highly enriched in the stroma of cancer tissues and produced mainly by CAFs. We have identified TGF-β3 by screening a panel of cytokines as the key regulator of POSTN expression in tumor microenvironment. In HNCs, the CAF-derived POSTN directly accelerated cancer cell proliferation, migration and invasion and indirectly modulated a tumor-supportive microenvironment in the primary and metastatic tumors, thereby resulting in the colonization initiation and metastasis of the tumor. As a result, POSTN may be an effective biomarker to predict the metastasis and a potential therapeutic target for HNCs.

## Materials and Methods

### Ethics statement

The tissue samples were collected from the Oral Cancer Tissue Bank of Shanghai affiliated to Ninth People’s Hospital of the Shanghai Jiao Tong University School of Medicine. The experiments were approved by the Clinical Research Ethics Committee of Ninth People’s Hospital, Shanghai Jiao Tong University School of Medicine. All methods were carried out in accordance with the approved guidelines of Medical Graduate School of Shanghai Jiao Tong University and the researches were implemented with reference to the provisions of the Helsinki Declaration of 1975. All the patients involved in this study signed written informed consent in accordance with the institutional guidelines.

### Patients and specimens

Twenty-nine pairs of tumor and adjacent normal tissues, seventy-three cases of tumor tissues and ninety-two cases of adjacent normal tissues were excised macroscopically from HNC patients who were diagnosed with primary HNC and underwent initial surgical resection in the Department of Oral and Maxillofacial-Head and Neck Oncology, Ninth People’s Hospital, Shanghai Jiao Tong University School of Medicine. In addition, the patients who provided the seventy-three tumor samples also provided other clinical information, and thirty-three of these patients had lymph node metastasis. In this study, tumor pathological differentiation and grade were determined according to standard criteria^52^ and the clinical tumor stage was assessed according to the TNM (tumor-node-metastasis) classification system of malignant tumors^53^.

### Cell cultures

The human HNC cell lines HN4, HN6, HN13 and HN30 were obtained from the National Institutes of Health^54^,^55^. The rat oral squamous cell carcinoma cell lines Rca-B and Rca-T were purified from tumor tissues induced by adding 4-nitroquinoline-1-oxide into rats’ drinking water^56^,^57^. CAFs and normal fibroblasts (NFs), isolated from tumor and adjacent normal tissues of HNC patients by primary culture, were tested for the presence of CAF-specific markers (α-SMA, FAP) and were selected to have less than 10% cytokeratin-positive cells. All these cells were cultured in Dulbecco’s modified Eagle’s medium (DMEM; GIBCO-BRL, USA) supplemented with 10% fetal bovine serum (FBS; GIBCO-BRL, USA), 100 units/mL penicillin and 100 μg/mL streptomycin at 37 °C in a humidified 5% CO_2_ atmosphere. Normal primary head and neck epithelial cells were cultured in keratinocyte serum-free medium (KSF; GIBCO-BRL, USA) with 0.2 ng/mL recombinant epidermal growth factor (rEGF; Invitrogen, USA).

### RNA extraction and real-time PCR analysis

Total RNA was extracted with TRIzol Reagent (Invitrogen, USA) according to the manufacturer’s protocol and reverse transcribed into cDNA using the PrimerScript RT reagent Kit (Takara, Japan). All the real-time PCR reactions were performed using an ABI 7300 real-time PCR system (Life Technologies, USA) and the SYBR Premix Ex Taq reagent kit (Takara, Japan). The reaction was performed according to the manufacturer’s instructions. The mRNA expression was quantified using delta delta CT method, and β-actin served as the internal control. The PCR primers are listed in Supplementary Table S1.

### Semi-quantitative RT-PCR analysis

One microliter of each RT reaction mixture was subjected to PCR amplification with EX Taq DNA polymerase (Takara, Japan). The PCR primers are listed in Supplementary Table S1. An initial denaturation step was performed for 5 minutes at 95 °C, and 36 cycles were performed with the following PCR program: denaturing at 95 °C for 30 seconds, annealing at 65 °C for 30 seconds, elongating at 72 °C for 30 seconds, and being completed with a final extension step at 72 °C for 10 minutes. The amplification results were determined by electrophoresis. Ethidium bromide-stained bands were visualized using UV transillumination, and fluorescence intensity was quantified using the FR-200 system (FuRi, Shanghai, China).

### Western blot analysis

Cells or tissues were harvested in SDS lysis buffer (Beyotime, China), and cell lysates (10 μg) were electrophoresed through 10% polyacrylamide gels and transferred to a PVDF membrane. The blots were probed with a mouse monoclonal POSTN antibody [47] (Sigma, USA, 1:1 000), a rabbit polyclonal POSTN antibody [polyclonal] (Sigma, USA; 1:1 000), a mouse monoclonal α-SMA antibody [1A4] (Abcam, USA; 1:300) and a rabbit monoclonal FAP antibody [S9B] (Abcam, USA; 1:1 000). A mouse monoclonal β-actin antibody [ac-74] (Sigma, USA; 1:5 000) was used throughout as a loading control. Secondary antibodies (Sigma, USA; 1:10 000) were labeled with IR Dyes. Signals were observed using an Odyssey Infrared Imaging System (Biosciences, USA).

### Immunohistochemical analysis

Briefly, paraffin-embedded sections were deparaffinized in xylene, rehydrated through graded ethanols, submerged into EDTA buffer for heat-induced antigenic retrieval, blocked with 2% bovine serum albumin, incubated with primary antibodies at 4 °C overnight and developed using the DAKO ChemMate Envision Kit ⁄HRP (Dako-Cytomation, USA) followed by counterstaining with hematoxylin, dehydration, clearing and mounting with neutral gums. The protein expression was determined by randomly selecting three tumor cell areas of each specimen under the same conditions using Image-pro plus 6.0 software. Primary antibodies were used at the following dilutions: rabbit polyclonal POSTN antibody [polyclonal] (Sigma, USA; 1:200), mouse polyclonal Pan CK antibody [AE1/AE3] (Ascend, China; 1:100) and mouse monoclonal Vimentin antibody [V9] (Sigma, USA; 1:200). For the immunohistochemical staining of cell climb slides, cells were spread on slides, fixed with 4% paraformaldehyde, incubated with 0.1% Triton X-100 and blocked with 2% bovine serum albumin before being incubated with primary anti-body. Normal oral epithelial cells or tissues were used as the normal control and the primary antibody was substituted by PBS in the blank control.

### Confocal microscope analysis

Cells were grown on cover slips, fixed in 4% paraformaldehyde, permeabilized with 0.1% Triton X-100 and incubated with a 1:100 dilution of POSTN antibody [47] (Sigma, USA). The primary antibody was detected using goat-anti-Mouse-Alexa 488-conjugated antibody (Invitrogen, USA; 1:500). Cells were co-stained with 4′, 6-diamidino-2-phenylindole (DAPI; Invitrogen, USA; 1:300) to detect nuclei. Cells were observed and imaged using a TCS SP2 laser-scanning confocal microscope (Leica Microsystems, Germany).

### ELISA analysis

Culture media of the 1 × 10^6^ fibroblasts (NFs and CAFs) were collected separately after incubation for 48 hours and the supernatant POSTN concentration was measured with a ELISA kit (Boster, China) according to the manufacturer’s protocol. Briefly, 100 μL standard samples and supernatant (the culture media were diluted 10 times) were added into each microtiter plate well in triplicate and the plate was incubated at 37 °C for 90 minutes. Then, the liquid in each well was removed and 100 μL biotin-labeled antibody was added into each well directly. After incubation at 37 °C for 60 minutes, the wells were washed three times and 100 μL avidin-peroxidase complex (ABC) was added into each well. About 30 minutes later, the wells were washed five times and 90 μL tetramethyl benzidine (TMB) solution was added per well for colouration. Whereafter, the plate was sealed and incubated at 37 °C for 30 minutes. The reaction was stopped by adding 60 μL stop buffer and the absorbance (A)450 nm values were measured by a microplate reader (Biohit, Helsinki, Finland) immediately. The standard curve was plotted using the concentration of the standard samples as the horizontal axis and the absorbance (A)450 nm value as the vertical axis. The POSTN concentration of the culture media could be calculated from the standard curve.

### Co-culture assay

A co-culture assay was implemented using 24-well transwell units with 3 μm porosity polycarbonate filters (Merck Millipore, USA). NFs (1 × 10^4^ cells in 600 μL DMEM medium containing 5% FBS) were added into the lower chamber, and tumor cells (1-2 × 10^4^ cells in 300 μL DMEM medium containing 5% FBS) were seeded into the upper chamber. Experiments were performed after the cells were incubated for 72 hours. Cells of different cell types were collected for the experiments.

### Plasmid construction and the transient and stable transfection of constructs

To obtain the POSTN expression vector, the open reading frame (ORF)of human POSTN cDNA was cloned into the eukaryotic expression vector GV144 (Genechem, China). The full-length POSTN gene was amplified by a set of primers (5′-GTCCGGACTCAGATCTCGAGCTATGATTCCCTTTTTACCCATG-3′/5′-TATCTAGATCCGGTGGATCCTCACTGAGAACGACCTTCCCTTAATC-3′). The amplified product of the POSTN gene was purified, digested and ligated into the respective HindIII and EcoRI sites in the GV144 vector. For transient transfection, cells were transfected with the POSTN plasmid using the Lipofectamine™2000 (Invitrogen, USA) reagent according to the manufacturer’s instructions. To generate stable POSTN-expressing cell lines, cells were selected with G418 (600 μg/mL) for two weeks before experiments.

### Transfection of small interfering RNAs

To knock down the expression of POSTN in CAFs, two POSTN gene-specific short interfering RNAs (siPOSTN-1 5′- CCCAUGGAGAGCCAAUUAUTT-3′and siPOSTN-2 5′- CUCUGACAUCAUGACAACAAAUTT-3′) were used. For transient transfection, CAFs were transfected with siRNAs at 30% confluence using the Lipofectamine™2000 (Invitrogen, USA) reagent according to the manufacturer’s instructions. The POSTN knock down was confirmed by Western blot analysis and qPCR after 48 hours.

### Cell proliferation analysis

After starvation for 6 hours, tumor cells were seeded onto 96-well plates at a density of 1000 cells in each well in triplicate. The proliferation of tumor cells was examined using CCK-8 (Dojindo, Japan) assay.

For the proliferation of tumor cell co-culture with fibroblasts, NFs or CAFs were transfected with POSTN-expressing plasmid or POSTN-siRNA beforehand. After 24 hours, a total of 3 × 10^3^ fibroblasts and 2 × 10^3^ HNC cells stably transfected with luciferase cDNA in DMEM containing 5% FBS were seeded in triplicate into 24-well plates. During a 4-day co-culture period, the tumor cell growth was monitored daily by measuring luciferase activity using a Luciferase Assay System (Promega Corporation, USA).

### Transwell migration assay

To evaluate the effect of fibroblast-derived POSTN on the migration of cancer cells, NFs or CAFs were transfected with POSTN-expressing plasmid or POSTN-specific siRNA in advance. Twenty-four hours later, 1 × 10^4^ fibroblasts were seeded into 24-well plates in 700 μL DMEM containing 2% FBS and incubated for 48 hours. To examine the effect of recombinant human POSTN (rhPOSTN) on the migration of cancer cells, 700 μL DMEM containing 10% FBS and 100 ng/mL rhPOSTN was added into the lower chamber. A total number of 2 × 10^4^ tumor cells in 300 μL serum-free DMEM were placed in each transwell chamber (Merck Millipore, USA) and allowed to migrate towards the fibroblast-CM for 24 hours. The membranes were fixed with paraformaldehyde and stained with crystal violet. Cells on the upper surface of the filter were removed by wiping with a small cotton swab and the cells that migrated through the membrane were photographed and quantified using Image J software. All experiments were performed in triplicate and three images were processed per membrane.

### Wound-healing assay

Tumor cells were harvested and plated in 6-well plates and cultured to confluence in regular culture medium. The plates were scraped with a P200 tip (time 0), washed with PBS, incubated with serum-free DMEM, and pictures of 5 non-overlapping fields were taken at the 12 hours and 24 hours. The cells over the baseline were counted in 5 random × 100 fields under a light microscope. To verify if the soluble POSTN act on HNC cells by binding an integrin receptor, tumor cells were incubated with specific anti-αv (5 μg/mL) and anti-β3 (5 μg/mL) integrin antibodies together for 30 minutes beforehand. Primary antibodies used were shown as following: a rabbit polyclonal integrin αv antibody [polyclonal] (Boster, China); a rabbit polyclonal integrin β3 antibody [polyclonal] (Boster, China).

### Transwell invasion assay

As it described previously^58^, the transwell invasion assays were carried out with Matrigel (BD Biosciences, USA) coated on the upper surface of the transwell chamber. What was different was that cancer cells, added into the upper transwell chamber, were resuspended with the conditioned medium (CM) derived from fibroblasts. In addition, NFs or CAFs, transfected with POSTN-expressing plasmid or POSTN-specific siRNA, were incubated with serum-free DMEM for another 48 hours and the culture medium was collected as CM. For the effect of rhPOSTN on the invasion of cancer cells, cells were resuspended serum-free DMEM containing 100 ng/mL rhPOSTN. After incubation for 30 hours, cells that invaded through the membrane were fixed with paraformaldehyde and stained with crystal violet. Images were randomly captured of three nonoverlapping fields of the fixed cells, and the cells were counted using Image J software. All experiments were performed in triplicate.

### Experimental metastasis assay

All animal procedures were performed in accordance with guidelines approved by the Shanghai Jiao Tong University School of Medicine. Female 4-week-old athymic mice (Shanghai Laboratory Animal Center) were used for studies. Rca-T cells (2 × 10^6^ cells in 0.2 mL serum-free DMEN) with different treatment were injected intravenously into the lateral tail vein. The animals were sacrificed at 1 hour, 1 day, 5 days, 10 days, 15 days and 20 days after hydrodynamic injection with Rca-T cells. Murine lung samples were fixed in neutral-buffered formalin, embedded in paraffin, and cut into 4-μm sections.

### Xenograft tumor assay

The xenograft experiment was implemented in female BALB/C nude mice (4-weeks-old) (Shanghai Laboratory Animal Center). All animal procedures were performed in accordance with the guidelines approved by the Shanghai Jiao Tong University School of Medicine. A total of 1 × 10^6^ Rca-T cells, together with a certain proportion (30%) of different POSTN-expressing fibroblasts, in 100 μL serum-free DMEM were subcutaneously seeding into the left and right buttocks. The tumor volume was measured every third day by the following formula: tumor volume = length × width × width/2, as previously noted^59^. The tumor growth curve was plotted using the seeding day as the horizontal axis and the tumor volume as the vertical axis.

### Statistical analyses

All statistical calculations were performed using SPSS 13.0 statistical software. Student’s *t*-test and one-way ANOVA were used to compare the means of 2 groups or more. Mann-Whitney U-test and Kruskal–Wallis test were used to analyze the data of the associations between POSTN mRNA levels and clinical parameters due to the abnormality and heterogeneity of the samples. Linear regression was applied to the correlation analysis between POSTN and TGF-β expression levels. All tests were two-sided and *p* values <0.05 was considered to be significant.

## Additional Information

**How to cite this article**: Qin, X. *et al.* TGFβ3-mediated induction of Periostin facilitates head and neck cancer growth and is associated with metastasis. *Sci. Rep.* **6**, 20587; doi: 10.1038/srep20587 (2016).

## Supplementary Material

Supplementary Information

## Figures and Tables

**Table 1:** Associations between *POSTN* mRNA levels and clinical parameters (N = 73)

Characteristic	No. of Patients		*POSTN* △Ct^a^Mean ± SD	*Non-parametric test value*	*P value*
	No.	%			
Age, years					
≥60	30	41.1	0.90 ± 1.84	*Z = −0.285*	*0.797*
<60	43	58.9	0.91 ± 1.97		
Gender					
Male	50	68.5	0.78 ± 1.92	*Z = −0.796*	*0.426*
Female	23	31.5	1.18 ± 1.89		
Smoking history					
Nonsmoker	38	52.1	0.72 ± 1.91	*Z = −0.939*	*0.348*
Smoker	35	47.9	1.10 ± 1.91		
Alcohol history					
Nondrinker	48	65.7	0.87 ± 1.89	*Z = −0.337*	*0.736*
Drinker	25	34.3	0.97 ± 1.98		
T stage					
T1	7	9.6	0.97 ± 1.61	*H = 3.501*	*0.321*
T2	29	39.7	0.54 ± 1.64		
T3	18	24.7	1.56 ± 1.87		
T4	19	26.0	0.82 ± 2.35		
Metastasis (N stage)					
pN+	33	45.2	1.42±1.74	*Z = −2.361*	*0.018*
pN-	40	54.8	0.48±1.95		
TNM stage					
I	7	9.6	1.12±1.54	*H = 3.015*	*0.389*
II	22	30.1	0.37±1.68		
III	18	24.7	1.33±1.86		
IV	26	35.6	0.99±2.18		
Pathological differentiation					
Well	32	43.8	1.28±1.74	*Z = −1.857*	*0.063*
Moderately/poorly	41	56.2	0.61±2.00		
Disease Site					
Tongue	34	46.6	1.34±2.06	*H = 5.008*	*0.286*
Gingival	9	12.3	0.36±1.89		
Cheek	9	12.3	0.56±2.03		
Floor of Mouth	14	19.2	0.23±1.52		
Oropharynx	7	9.6	1.42±1.45		
Recurrence					
Yes	13	17.8	0.74±1.83	*Z = −0.418*	*0.676*
No	60	82.2	0.94±1.94		

Abbreviations: POSTN, osteoblast-specific factor 2; SD, standard deviation; T, tumor stage; N, lymph node stage; TNM stage, tumor-node-metastasis stage.
