# Supplementary material for: TGFβ3-mediated induction of Periostin facilitates head and neck cancer growth and is associated with metastasis
Source: Sci Rep. 2016 Feb 9;6:20587. doi: 10.1038/srep20587 (PMC4746667; doi:10.1038/srep20587)
Supplement: Supplementary Information [file srep20587-s1.pdf]

TGFβ3-mediated induction of Periostin facilitates head and neck cancer growth and is associated with metastasis

Xing Qin[1,2#], Ming Yan[1,2#], Jianjun Zhang[1,2], Xu Wang[1,2], Zongze Shen[1,2], Zhongjing Lv[1,2], Zhihui Li[1,2], Wenyi Wei[1,3] and Wantao Chen[1,2]*

Supplementary files

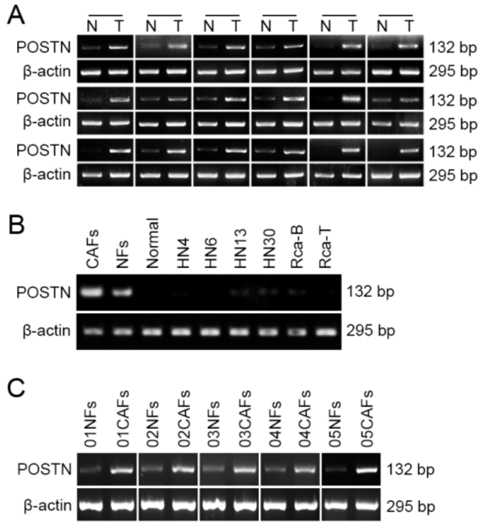

Supplementary Figure S1. The mRNA levels of POSTN in different HNC associated samples. (A) Representative results of POSTN mRNA levels from 18 pairs of HNC tissues and their paired adjacent normal tissues. (B) The translational statuses of POSTN were determined in CAFs and NFs, 6 representative HNC cell lines and normal oral epithelial cells (titled normal) using semi-quantitative RT-PCR. (C) POSTN mRNA levels in 5 pairs of NFs and CAFs were measured by semi-quantitative RT-PCR.

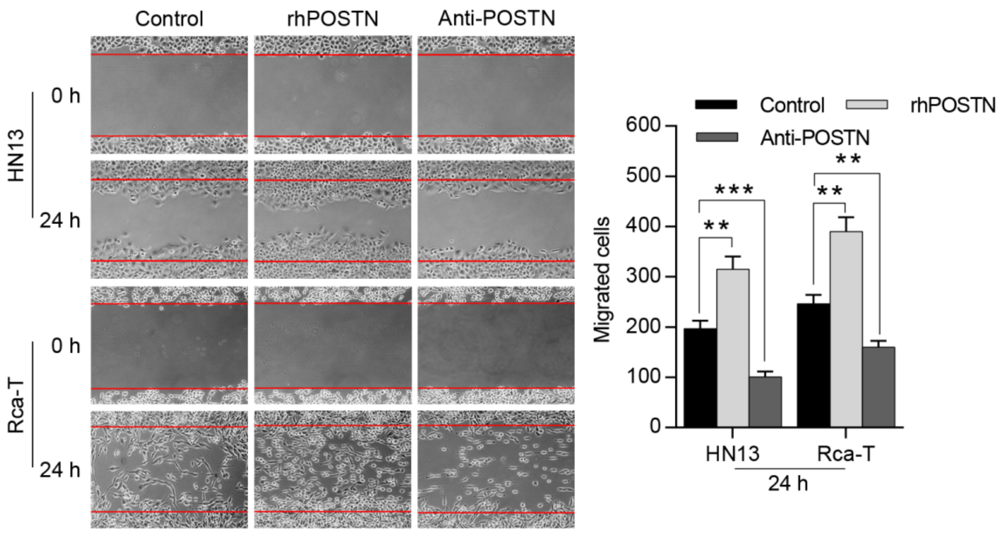

Supplementary Figure S2. Recombinant human POSTN (100 ng/mL) promoted the migration of HNC cells (HN13 and Rca-T), while neutralization of POSTN inhibited the migration of HNC cells (HN13 and Rca-T).

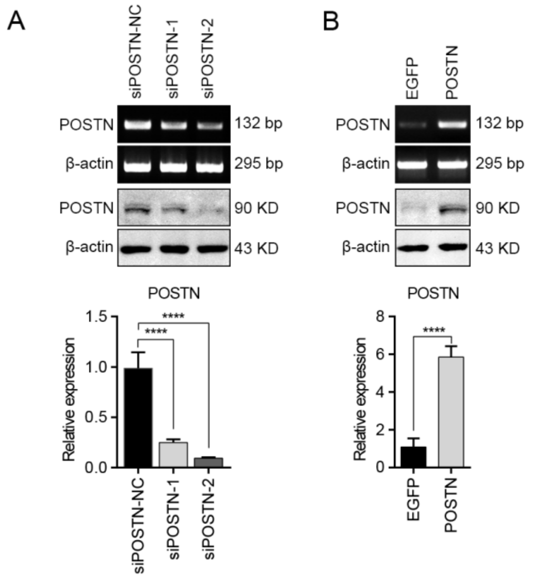

Supplementary Figure S3. POSTN expression in fibroblasts after POSTN knockdown or overexpression. (A) The results of semi-quantitative RT-PCR,

western blot analysis and real-time PCR showing POSTN expression levels in CAFs after transfection of siRNA targeting POSTN. (B) The results of semi-quantitative RT-PCR, western blot analysis and real-time PCR showing POSTN expression levels in NFs after POSTN expression vector (POSTN) transfection. ****$p<0.0001$.

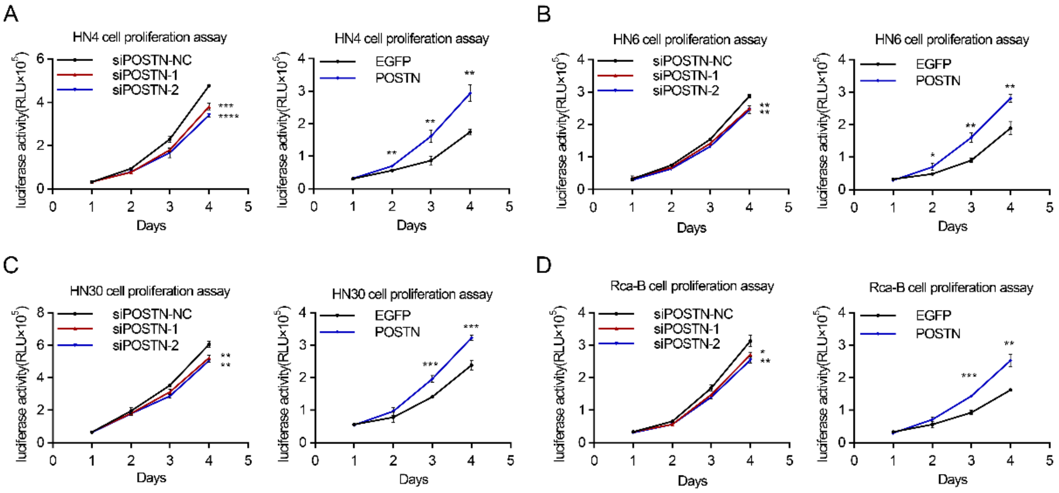

Supplementary Figure S4. Functional analysis of POSTN effects on HNC cell proliferation. (A-D) POSTN knockdown in CAFs partially inhibited the growth of HNC cells (HN4, HN6, HN30 and Rca-B), while POSTN overexpression in NFs facilitated the proliferation of HNC cells (HN4, HN6, HN30 and Rca-B). * $p<0.05$;** $p<0.01$; *** $p<0.001$; **** $p<0.0001$.

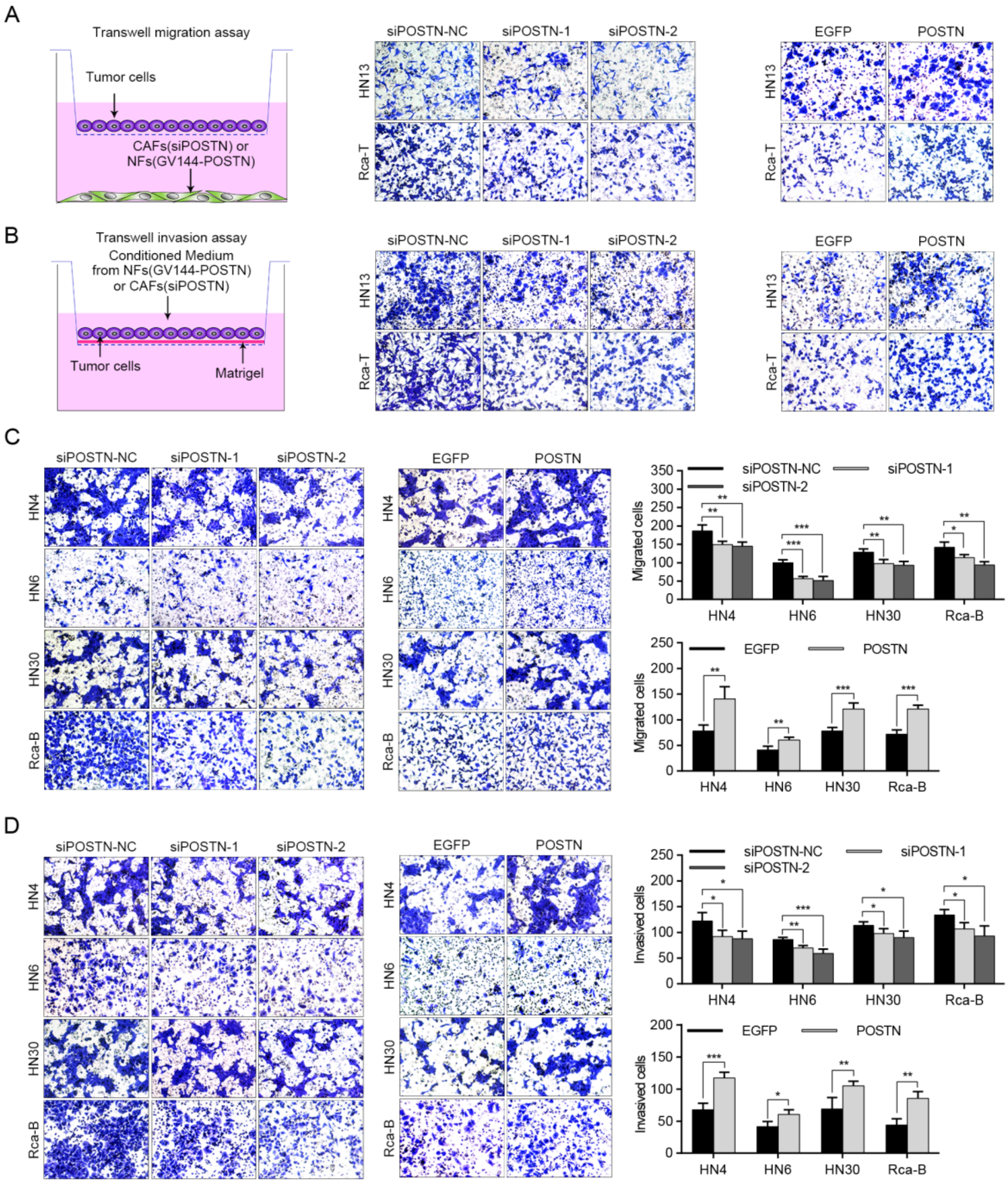

Supplementary Figure S5. Functional analysis of POSTN effects on HNC cell migration and invasion. (A, B) Models illustrating the methods of transwell migration and invasion assay. POSTN knockdown in CAFs inhibited the migratory and invasive abilities of HN13 and Rca-T cells. POSTN overexpression in NFs increased the migration and invasion of HN13 and Rca-T cells. (C, D) POSTN knockdown in CAFs inhibited the migratory and invasive abilities of HNC cells (HN4,

HN6, HN30 and Rca-B), while POSTN overexpression in NFs increased the migration and invasion of HNC cells (HN4, HN6, HN30 and Rca-B). * p<0.05;** p<0.01; *** p<0.001.

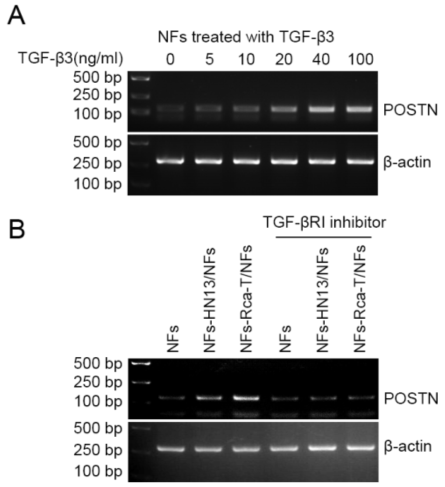

Supplementary Figure S6. TGF-β3 induced POSTN expression in NFs. (A) TGF-β3 induced POSTN expression in NFs in a dose-dependent manner and the mRNA expression by semi-quantitative RT-PCR were measured at the indicated concentrations. (B) Blocking of TGF-β3 with the inhibition of type Ⅰ TGF-β receptor (SB431542, 10 μM) abrogated the induced-POSTN expression in NFs after the co-culture of NFs and HNC cells. POSTN mRNA levels were determined in NFs using semi-quantitative RT-PCR.

Supplementary Table S1. The primers used for real-time PCR and semi-quantitative RT-PCR analyses.

Genes	Primer sequences
Human POSTN	forward 5'-GCACTCTGGGCATCGTGGGA -3'
	reverse 5'-AATCCAAGTTGTCCCAAGCC -3'
Human SPP1	forward 5'-GAAGTTTCGCAGACCTGACAT -3'
	reverse 5'-GTATGCACCATTCAACTCCTCG -3'
Human TGF-β1	forward 5'-TCGCCAGAGTGGTTATCTTTTG -3'
	reverse 5'-AGGAGCAGTGGGCGCTAAG -3'
HumanTGF-β2	forward 5'- ATCCCGCCCACTTTCTAC -3'
	reverse 5'-GCTCAATCCGTTGTTCAGG -3'
HumanTGF-β3	forward 5'-GCCCTTGCCCATACCTCCGC-3'
	reverse 5'-CGCAGCAAGGCGAGGCAGAT -3'
Human IL-4	forward 5'- ACATTGTCACTGCAAATCGACACC -3'
	reverse 5'- TGTCTGTTACGGTCAACTCGGTGC -3'
Human IL-13	forward 5'- GCAATGGCAGCATGGTATGG -3'
	reverse 5'- AAGGAATTTTACCCCTCCCTAACC -3'
Human BMP2	forward 5'- ACTCGAAATTCCCCGTGACC -3'
	reverse 5'- CCACTTCCACCACGAATCCA -3'
Human PDGF-bb	forward 5'- CAGCGCCCATTTTTCATTCC -3'
	reverse 5'-GTTTTCTCTTTGCAGCGAGGC-3'
Human β-actin	forward 5'- TCACCCACACTGTGCCCATCTACGA -3'
	reverse 5'- CAGCGGAACCGCTCATTGCCAATGG -3'
Rat POSTN	forward 5'-ATCTTCCTCAGCCTCCTC -3'
	reverse 5'-CCCAATCAGAATCTCCCT -3'
Rat TGF-β1	forward 5'- AATTCCTGGCGTTACCTTGGT -3'
	reverse 5'- GAAGGGTCGGTTCATGTATG -3'

Rat TGF-β2	forward 5’- TGCCATCCCGCCCACTTTCTAC -3’
	reverse 5’- CAATCCGTTGTTCAGCCACTCT -3’
Rat TGF-β3	forward 5’- CACAGTCCGCTACTTCGTC -3’
	reverse 5’- CCCTAATGGCTTCCACCCTC -3’
Rat IL-4	forward 5’-CCACGGAGAACGAGCTCATC -3’
	reverse 5’-ACCGAGAACCCCAGACTTGTT -3’
Rat IL-13	forward 5’-CTCGCTTGCCTTGGTG -3’
	reverse 5’-TGATGTTGCTCAGCTCCTC -3’
Rat BMP2	forward 5’-CCTATATGCTCGACCTGTAC -3’
	reverse 5’-CCCACTCATTTCTGAAAGTTC -3’
Rat PDGF-bb	forward 5’- GGAGCTTCACAGAGGACTGG -3’
	reverse 5’-GATCTGGGTGCCATGAGAGT -3’
Rat β-actin	forward 5’-ACGTTGACATCCGTAAAGACC -3’
	reverse 5’-AACGCAGCTCAGTAACAGTCC -3’
